# Caffeine Ingestion With or Without Low-Dose Carbohydrate Improves Exercise Tolerance in Sedentary Adults

**DOI:** 10.3389/fnut.2019.00009

**Published:** 2019-02-12

**Authors:** Namrita Kumar, Gordon L. Warren, Teresa K. Snow, Melinda Millard-Stafford

**Affiliations:** ^1^School of Applied Physiology, Georgia Institute of Technology, Atlanta, GA, United States; ^2^Department of Physical Therapy, Georgia State University, Atlanta, GA, United States

**Keywords:** nutrition, endurance, perceived exertion, fatigue, lactate threshold

## Abstract

Caffeine (CAF) and carbohydrate (CHO) ingestion delay fatigue during prolonged exercise; however, this is primarily documented in endurance trained (ET) athletes. Our purpose was to determine if these ergogenic aids are also effective to improve exercise tolerance in age-matched sedentary (SED) adults. Using a double-blind crossover design, ET and SED (*n* = 12 each group) completed four exercise trials consisting of 30 min cycling at standardized matched work rates 10% below lactate threshold (MOD-EX) followed by a time to fatigue (TTF) ride at individually prescribed intensity of 5% above lactate threshold. After standardized breakfast, the following drink treatments were given before and throughout exercise: CAF (3 mg/kg of body mass, equivalent to 1.5 cups premium brewed coffee), low calorie CHO (LCHO) (0.4% solution, 2 g total CHO), CAF+LCHO, and artificially-sweetened placebo (PLA). SED and ET had similar perceived exertion (RPE) during MOD-EX and TTF (23.8 ± 3.1 and 24.1 ± 2.6 min in ET, SED, respectively). LCHO did not benefit exercise tolerance compared to PLA and was less effective (*p* < 0.05) compared to CAF+LCHO for all participants combined. Thus, the two CAF treatments were averaged, resulting in ~5% lower RPE (*p* < 0.05) and 21% longer TTF (26.3 ± 10.4 min) compared to the no-CAF (21.7 ± 9.9 min) treatments. Blood glucose and lactate were higher (*p* < 0.05) with CAF vs. no-CAF. SED and ET only differed in metabolic oxidation rates during exercise (higher overall fat oxidation with ET compared to SED). CAF reduces the perceived effort during exercise and increases the capacity for sedentary individuals, as well as trained athletes, to tolerate higher intensity exercise for greater duration; and, these benefits were not further enhanced by ingesting doses of low carbohydrate regularly during exercise.

## Introduction

Athletes ingest caffeine and carbohydrate to improve performance and delay fatigue during exercise (1–4). Fatigue is a complex phenomenon manifested by an increased perception of effort and/or the inability to sustain a task. Fatigue may be a contributing factor deterring sedentary individuals from physical activity (5). Whether a generalized fatigue prevents the undertaking of exercise or the acute discomfort/fatigue that directly results from exercise is the more relevant barrier remains unclear (6). In 2017, only 23.5% of adults in the U.S. met the 2008 Federal Guidelines for accumulating sufficient levels of moderate to vigorous physical activity (7). Therefore, any deterrent that influences physical activity behavior (e.g., fatigue) may adversely impact not only fitness but also a variety of other health outcomes (8). However, there is a knowledge gap regarding the appropriate nutritional strategies to mitigate fatigue in sedentary populations as most research, to date, has focused primarily on such strategies for athletic populations.

Caffeine (CAF), a well-documented ergogenic aid, reduces fatigue through a number of potential mechanisms: direct effect on muscle via increased mobilization of intracellular calcium and/or increased sensitivity (9), increased motor unit recruitment in the central nervous system (CNS) (10), and/or increased lipolysis and fat oxidation with associated glycogen sparing (8, 11–13) although the latter has recently become less accepted (14). CAF also has neuromodulatory effects in the brain enhancing task persistence and determination likely through adenosine inhibition (10, 15), thus, allowing individuals to perform more work at the same effort or perceive the same exercise intensity as more tolerable and/or less painful (1, 2, 16, 17).

Carbohydrate (CHO) also enhances exercise capacity (4) by maintaining blood glucose and enhancing CHO oxidation (3). Although 30–60 g/h of CHO has been recommended, lower doses (<15 g/h) may be effective (18), even when glycogen status is not limiting and blood glucose well maintained (3). A central mechanism of CHO may act by stimulating CHO-sensitive oral cavity receptors (e.g., CHO mouth rinse) that signal brain areas modulating behavioral response to rewarding stimuli; possibly increasing motivation and allocation of neural resources to enhance exercise performance (19, 20).

Studies investigating CAF and CHO effects on endurance capacity have been limited primarily to highly-trained and recreationally-active individuals (1, 4, 21). Individuals of higher fitness (VO_2max_) may attain greater benefit from CAF (2) based on the inverse correlation between exercise perceived effort and VO_2max_. Yet, few studies have investigated effects of CAF (22–24) or CAF in a low-content CHO drink (25, 26) on exercise capacity in a true sedentary population; and, of these, the results regarding exercise tolerance appear mixed (27). Moreover, effects of CAF based on fitness level are rarely investigated within the same study (25) and in this lone study only endurance-trained athletes (vs. less active individuals) improved time trial performance with CAF. Training status might influence the effectiveness of caffeine since metabolic adaptations from training would be absent in sedentary individuals (e.g., less ability to utilize fat oxidation) (9). Moreover, since CAF may elicit insulin resistance under sedentary conditions (28), further research is warranted to better understand if CAF (with or without CHO) benefits a healthy but sedentary population to improve exercise tolerance.

Our aim was to determine if exercise tolerance is improved in sedentary similar to trained individuals with CAF, and whether adding “low” CHO (LCHO) provides additional benefit. We hypothesized CAF and CAF+LCHO ingestion would both improve exercise tolerance independent of fitness level; and, that each nutritional component alone would improve measures of fatigue compared to a placebo (PLA). Further, we expected CAF+LCHO would only be more beneficial than CAF alone, if LCHO provided benefit compared to PLA.

## Methods

### Participants

Twelve ET and 12 healthy SED males (*n* = 10) and females (*n* = 2) volunteered to participate in this study. Participants provided written informed consent prior to the study as approved by the Institutional Review Board. ET and SED were matched pairwise by sex, age (within 3 year), body mass, and BMI (within 2 kg/m^2^). Physical characteristics of participants are presented in Table 1. ET were recruited from the local cycling, triathlon, and cross-country clubs or competitive teams, reporting >360 min/wk of exercise training (averaging 753 ± 364 min/week). SED were college students not engaging in regular exercise with daily behaviors characterized by only low energy expenditure (mostly of sitting time, limited to low intensity walking <2.5 metabolic equivalents). Time spent in transport or non-exercise physical activity (i.e., walking or riding a bike to class or work) was also validated using the Core and Expanded Physical Activity STEPS version 2.0 Instrument (29). SED and ET were similar in average non-exercise physical activity (130 ± 136 and 108 ± 106 min/wk). Inclusion criteria for SED were also verified based on maximal aerobic capacity (<45 and <40 ml/kg-min for men and women, respectively). Table 1 indicates the groups differed (*p* < 0.05) in % body fat, VO_2peak_, %VO_2peak_ at lactate threshold (LT). Based on relative VO_2max_ classifications and cycling training history (30), ET met the criteria as Performance Level 3 and SED as Performance Level 1. This unified classification system by DePauw et al. (30) was used since often participant descriptions are arbitrarily designated as “sedentary vs. physically active” or “untrained vs. trained.” Level 1 corresponded to the lowest activity level based on a 5 point scale with Level 5 classified as elite level endurance cyclists.

**Table 1:** Mean (±SD) physical characteristics of participants.

	**Endurance-trained (*n* = 12)**	**Sedentary (*n* = 12)**
Age (yr)	27.7 ± 5.5	26.8 ± 7.0
Body mass (kg)	72.2 ± 8.3	72.7 ± 11.4
BMI (kg/m^2^)	23.1 ± 2.1	23.7 ± 2.9
Body fat (%)	14.1 ± 5.8	22.6 ± 10.1^*^
VO_2peak_ (ml/kg-min)	56.5 ± 9.0	36.9 ± 7.9^*^
Watts at VO_2peak_ (Wmax)	319 ± 69	217 ± 62^*^
Blood lactate at VO_2peak_ (mmol/L)	7.6 ± 1.9	9.7 ± 1.1^*^
%VO_2peak_ at LT	75.7 ± 10	67.5 ± 9.1^*^
%Wattmax at LT	69.4 ± 18	63.5 ± 4.5
RPE at LT	13.0 ± 1.7	14.0 ± 1.4
%HRmax at LT	82.3 ± 7.4	83.7 ± 8.2
Blood lactate at LT (mmol/L)	2.4 ± 1.0	4.2 ± 1.1^*^

* p < 0.05 indicates significant difference vs. endurance-trained participants. LT indicates values at lactate threshold

All participants completed a health-history screening questionnaire and habitual caffeine intake questionnaire (31) to ensure they met all inclusion criteria. Participants were excluded if either naïve to caffeine usage or above a “high” range (32) (i.e., consuming > 500 mg/d) based on reference population data.

### Research Design

A double-blind, placebo-controlled, repeated measures crossover design was used and treatment order was assigned using a Latin Squares method. All participants served as their own control performing four trials (LCHO, CAF, CAF+LCHO, and PLA).

### Preliminary Testing

Anthropometric measures including body mass, height, and body composition (% body fat) using the GE Lunar Prodigy Dual X-Ray Absorptiometry (DXA) scanner (GE Healthcare, Hatfield, United Kingdom) were taken. In a thermoneutral environment, participants completed a ramped exercise protocol using two- min stages with increases of 25–50 W until volitional fatigue on an electrically-braked ergometer (Lode Excalibur Sport, Lode, Groningen, The Netherlands) to determine VO_2peak_. Gas exchange data were obtained with the ParvoMedics True One 2,400 metabolic cart (ParvoMedics, Sandy, UT). Heart rate (HR) and rating of perceived exertion (RPE) (33) were recorded every minute. We ensured a maximal effort was obtained in ET and SED based on similar respiratory exchange ratio (RER) (1.16 ± 0.05 and 1.15 ± 0.11) and RPE (18.3 ± 1.7 and 17.7 ± 2.4). Blood lactate (HLa) was measured two min post-exercise using a 0.3 μL blood sample obtained from the ear lobe (Lactate Pro LT-1710 analyzer, Arkray, Japan). During the subsequent experimental exercise tests, blood samples were drawn from a lanced fingertip into a heparinized capillary tube (Microvette® CB300, Sarstedt AG&Co., Numbrecht, Germany) to measure both blood glucose and HLa (YSI Life Sciences, Inc. Yellow Springs, OH).

In order to assign comparable exercise workloads for ET and SED, each participant's lactate threshold (LT) was also determined using a ramped cycling protocol. Participants began cycling at 50 W and power output was increased by 25–50 W every three min until reaching a RPE of 16–17 (“Hard to Very Hard”). HLa was measured at baseline and 2 min into each stage. Each individual's LT was determined using the DMax method (34) in order to calculate moderate (10% < LT) and vigorous (5% > LT) workloads for the moderate exercise (MOD-EX) and time to fatigue (TTF) portions of the experimental protocol, respectively.

Prior to each experimental trial, participants were provided pre-test instructions (to obtain adequate sleep, hydration, and refrain from caffeine intake and physical activity the day prior to test sessions). Because ET were engaging in regular endurance training, we requested they refrain from strenuous training or racing in the three days prior to experimental sessions.

### Treatment Ingestion

Fruit punch solutions were manufactured by Glaceau (New York, NY, USA) and shipped in de-identified containers with either CAF (0.34 mg/ml), LCHO (3.6 g/L), CAF+LCHO, or PLA (artificially sweetened with aspartame) to maintain the double blind design. Fluid volumes were administered based on individual's body mass to provide 3 mg/kg CAF, resulting in 464 ± 85 ml of fluid. The treatment beverage (all but 100 ml) was given 40 min prior to exercise with the remainder ingested as 25 ml boluses (3 times throughout MOD-EX and 25 ml at the start of TTF) without mouth rinsing. An energy bar (PowerBar® Harvest Energy, PowerBar USA, Florham Park, NJ) was consumed along with the treatment beverage, providing 250 kcal (5 g fat, 43 g carbohydrate, 9 g protein). Thus, the total CHO dose combined with the initial beverage bolus was 43 g in CAF and PLA trials and 44.7 ± 0.3 g in CAF+LCHO and LCHO trials.

### Experimental Protocol

**Figure 1:**
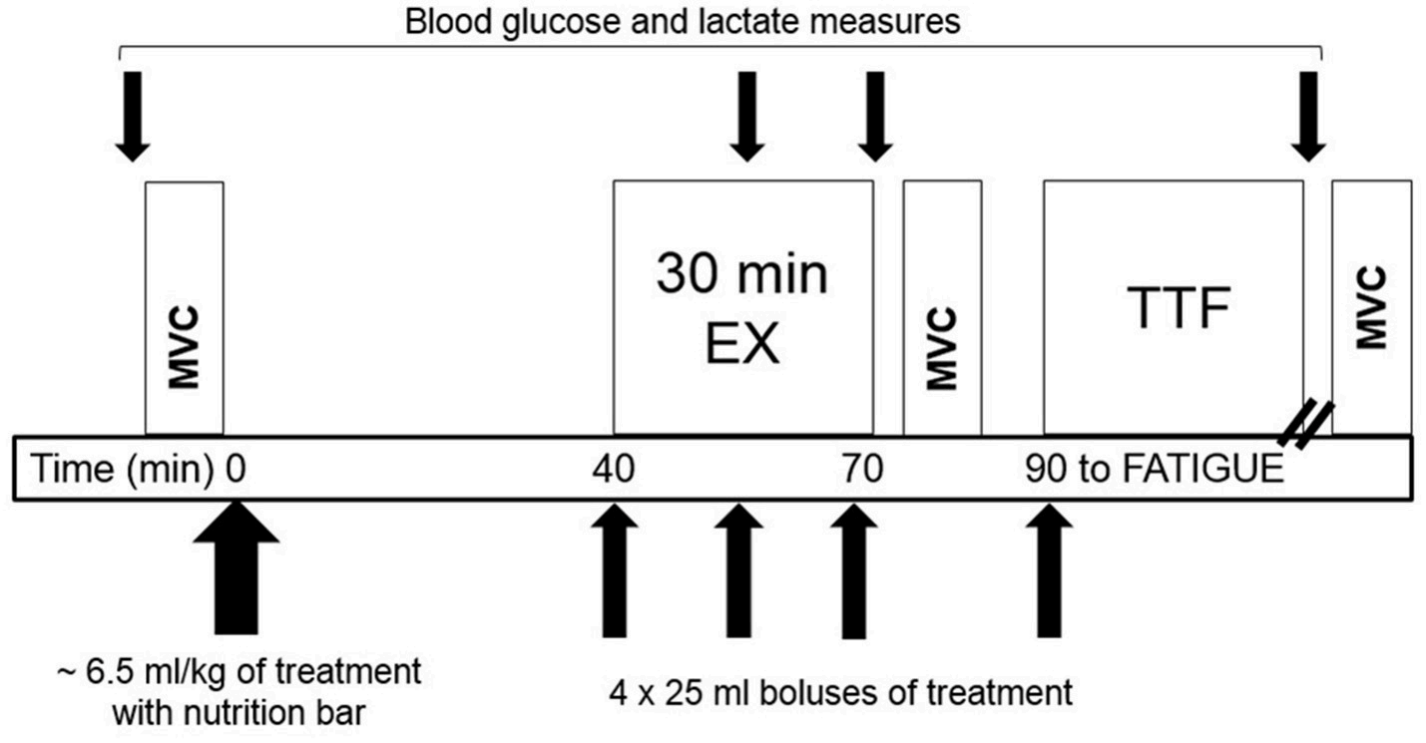
Schematic of the test protocol for the four drink trials (one initial 6.5 ml/kg bolus and four additional 25ml boluses during exercise) consisting of moderate-intensity cycling (EX) followed by cycling to volitional fatigue (TTF) with timing of Maximum Voluntary Contractions (MVC).

The next four visits were each separated by at least 7 d and scheduled at the same time of morning following an overnight fast. Before each visit, participants refrained from exercise for 24 h and caffeine for 12 h, confirmed with a brief 24 h history questionnaire at the beginning of each trial. The schematic of the test protocol is illustrated in Figure 1. Upon arrival to the lab, participants completed a 24 h diet recall. After the first visit, the 24 h diet recall was copied and returned to the participant to use as a guide for replicating 24 h dietary intake before subsequent visits.

In order to give sufficient time for CAF to reach peak concentration, the exercise protocol began 40 min after treatment ingestion. During MOD-EX, participants warmed up at 50 W (SED) or 100–150 W (ET) for 5 min prior to cycling continuously for 25 min at the individual's predetermined workload. VO_2_ and RER were measured during the last 3 min of each 10-min interval. Total-body CHO and fat oxidation were calculated from gas exchange data based upon VO_2_ and non-protein RER (35). RPE and HR were assessed every 5 min during MOD-EX and each min during TTF. Blood glucose and HLa were measured at 15 and 30 min and after the TTF. After a 10 min break, participants began TTF at the predetermined workload. The point of fatigue was defined as the participant voluntarily stopping exercise due to fatigue and/or the inability to maintain a minimum cycling cadence of 40 rpm. Participants received no information on time elapsed, HR, or provided any external motivation.

Isometric strength of the right knee-extensor muscles was measured during maximal voluntary contractions (MVC) on a modified leg-extension/curl machine (model NT-1220, Nautilus Fitness Products, Louisville,CO) connected to a force transducer (model SBO-300-T, Transducer Techniques, Temecula, CA), using procedures previously described (31, 36). MVC strength was determined at baseline prior to each experimental session and within 5 min after exercise (MOD-EX and TTF).

## Statistical Analyses

Data are reported as mean ± standard deviation (SD) and were analyzed using SPSS 17.0 (Chicago, IL). Three-way (group x treatment x time) mixed model ANOVA (group as the between-subject factor and treatment as the within-subject factor with repeated measures over time) was used to examine differences in substrate oxidation, RPE and physiological variables and a one-way (within factor treatment) ANOVA for TTF and overall RPE for all participants combined as one group. The Greenhouse-Geisser correction was used to account for the sphericity assumption of unequal variances across groups. If a significant F ratio was obtained, Bonferroni *post hoc* test was used to detect significant differences in pairwise comparisons. Pearson product moment correlations were used to determine if habitual caffeine intake was associated with TTF. Statistical significance was considered achieved with an alpha level of *p* < 0.05.

Sample size was based on a priori power analysis completed with G^*^Power 3.1. Based on the ES of 0.7 for CAF compared to PLA on endurance capacity and RPE (1, 2), a total sample size of 24 yielded statistical power of 0.8 (rho = 0.5, alpha level = 0.05, two groups, four treatments). In the study by Astorino et al. (25) with eight participants in each of two groups (active vs. endurance-trained), CAF improvements in time trial performance were only observed in the endurance-trained group, suggesting the current study was adequately powered to find differences in caffeine effects across groups with different fitness levels. The ES between the endurance-trained and active groups' time trial performance (25) when ingesting caffeine (on the second of two caffeine trials) was calculated to be 1.8, resulting in a power of 0.92 with 8 participants per group.

## Results

### Exercise Workloads

When comparing ET and SED, the %Watt maximum, RPE, and %HR maximum (%HRmax) were not different at LT; however, %VO_2_peak and HLa at LT was different (*p* < 0.05) between groups (Table 1). ET cycled at a higher VO_2_ uptake during MOD-EX (*p* < 0.001) (36.2 ± 4.8 ml/kg-min) compared to SED (25.7 ± 4.8 ml/kg-min).

### Low-CHO Treatment

Compared to PLA, LCHO did not result in any significant differences in HR, RPE, or muscular strength. In addition, LCHO did not affect blood glucose, HLa, or substrate utilization. RER during MOD-EX was 0.954 ± 0.044, 0.959 ± 0.054, 0.953 ± 0.049, and 0.950 ± 0.044 for CAF+LCHO, CAF, PLA, and LCHO respectively, *p* > 0.05. The addition of LCHO to CAF also did not elicit significant differences compared to CAF alone. Thus, the remaining results are presented as comparisons between CAF (CAF and CAF+LCHO) vs. no-CAF treatments (LCHO and PLA) unless otherwise specified. This approach is also consistent with the previous study by Astorino et al. (25) comparing endurance trained vs. “active” individuals utilizing two caffeine drink trials (mixed with a low carbohydrate powder) to evaluate perceptual effects compared to placebo.

### Time to Fatigue

TTF (averaged across all trials) was similar (*p* = 0.94) in ET (23.8 ± 8.1 min) compared to SED (24.1 ± 11.3 min) and exercise time for all trials is presented in Table 2. There was a main treatment effect (*p* = 0.004) but no group x treatment interaction. TTF was significantly longer (*p* = 0.03) with CAF+LCHO by ~26% compared to LCHO. As shown in Table 2, this was likely attributable to the fact that ET had the worst overall performance in LCHO (14% lower time to fatigue than compared with Placebo). By comparison, SED had the worst overall performance with PLA (LCHO had a non-significant 5% longer time to fatigue). Both caffeine treatments had higher mean time to fatigue compared to the other two non-caffeinated treatments across ET and SED. When CAF and CAF+LCHO treatments were averaged to examine CAF effects, TTF was longer (*p* = 0.004) with CAF (26.3 ± 10.4 min) compared to no-CAF (21.7 ± 9.9 min) (ES = 0.45, *p* < 0.01). When all treatments with LCHO were averaged, TTF was not different (24.0 ± 10.7 min) from no-CHO (24.0 ± 9.4 min). TTF was not associated with participants' habitual caffeine intake during either the CAF trials (*r* = −0.18, *p* = 0.394) or no-CAF trials (*r* = 0.038, *p* = 0.861).

**Table 2:** Mean (±SD) minutes of cycling to volitional fatigue during time to fatigue trial (5% above LT).

**Treatment**	**Endurance-trained (*n* = 12)**	**Sedentary (*n* = 12)**	**All participants (*n* = 24)**
Caffeine (CAF)+LCHO	26.5 ± 9.6 (*d* = 0.4)	26.9 ± 15.6 (*d* = 0.38)	26.7 ± 12.6 (*d* = 0.39)
Caffeine (CAF)	26.0 ± 10.7 (*d* = 0.32)	25.6 ± 9.2 (*d* = 0.37)	25.8 ± 9.8 (*d* = 0.36)
Placebo (PLA)	22.8 ± 9.0	21.5 ± 12.8	22.1 ± 10.8
Low-Carbohydrate (LCHO)	19.8 ± 10.3 (*d* = −0.31)	22.5 ± 10.4 (*d* = 0.09)	21.2 ± 10.2^*^ (*d* = −0.1)
All treatments	23.8 ± 3.1	24.1 ± 2.6	24.0 ± 2.6

* *p < 0.05 indicates significantly lower compared to CAF+LCHO*.

*Effect size (Cohen's d) is treatment compared to PLA for all participants*.

### RPE

**Figure 2:**
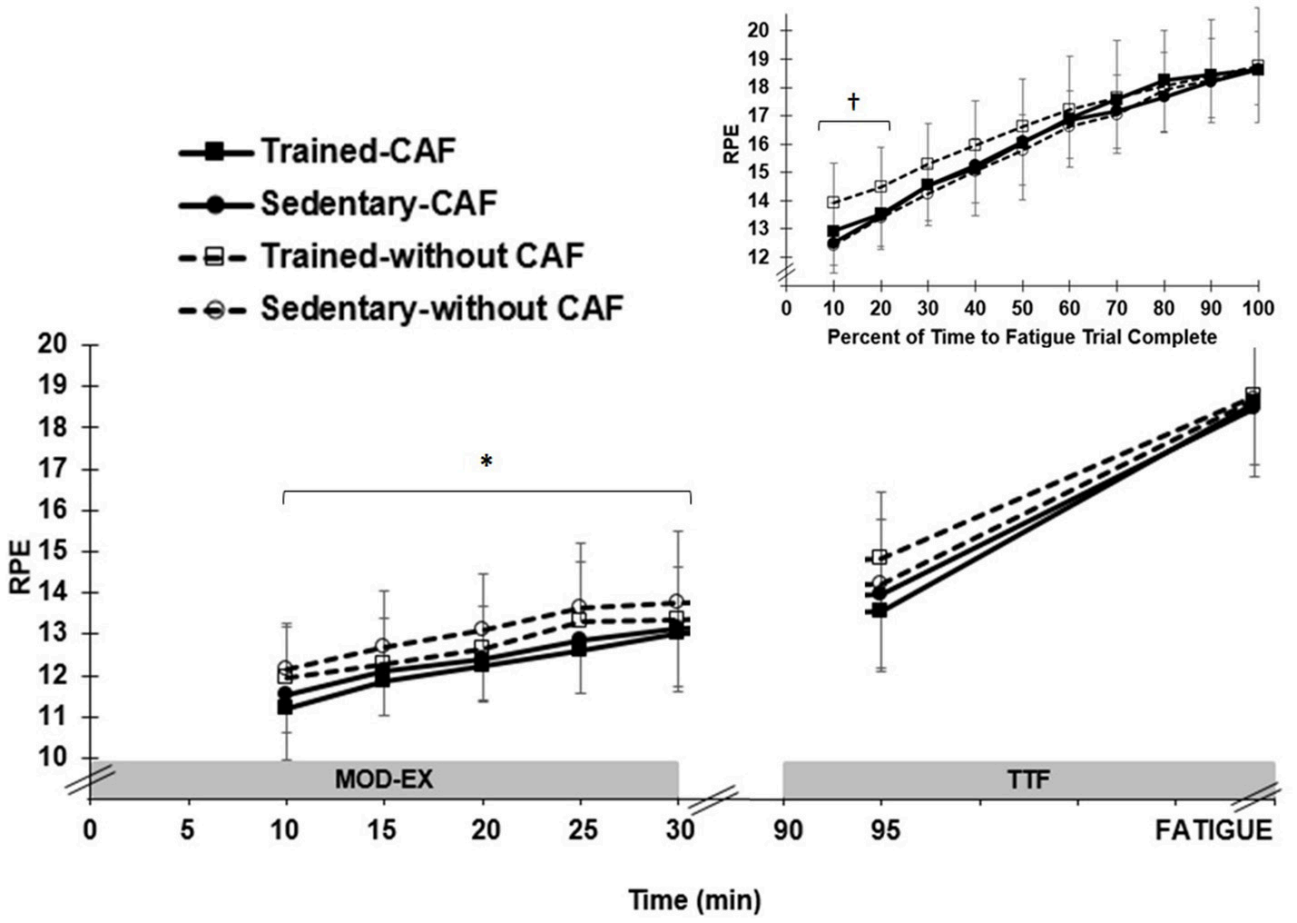
Mean (±SD) rating of perceived exertion (RPE) by group (trained and sedentary) and treatment (caffeinated CAF vs. no CAF) during moderate exercise (MOD-EX) and time to fatigue (TTF). *Significant (*p* < 0.01) lower RPE with CAF compared to no CAF. Upper right panel: RPE normalized by percentage of trial completed during TTF. ^†^Significantly higher (*p* < 0.05) RPE in trained participants without CAF during first 20% of TTF.

RPE averaged over 30 min MOD-EX was similar (*p* = 0.50) in SED (12.4 ± 1.2) and ET (12.0 ± 0.9) and increased significantly (*p* < 0.001) between 10 (11.7 ± 1.1) and 25 min (13.1 ± 1.2). For all participants, CAF resulted in ~5% lower (*p* = 0.005) overall RPE (11.9 ± 1.0) compared to no-CAF (12.5 ± 1.2) (Figure 2), but no treatment x time interaction (*p* = 0.18).

RPE was similar (*p* = 0.41) in SED (16.0 ± 1.0) and ET (16.4 ± 1.5) when averaged over time throughout the entire TTF. However, since all participants rode until exhaustion, a similar RPE is expected nearing the end of the TTF trial, as reported by Astorino et al. (25). Therefore, the % time in the TTF spent below an absolute perceived exertion score of “very hard” (16 on 20 point scale) was calculated on an individual basis (since times to fatigue were not uniform across participants and/or treatments). CAF extended (*p* = 0.003) the cycling duration by ~32% (14.8 ± 7.3 min) prior to participants rating a “very hard” effort compared to no-CAF (11.2 ± 5.5 min). When the duration of each TTF was normalized for % of trial completed, there was no main effect of CAF (*p* = 0.30), but a significant three-way interaction between group × treatment x time (*p* < 0.001). Compared to no-CAF, CAF resulted in a lower (*p* < 0.05) RPE early in the trial (first 20% of total cycling time) for ET, but this effect was not observed in SED (Figure 2).

### Substrate Utilization

Substrate utilization and energy expenditure (EE) during MOD-EX were measured. Total EE was higher (*p* = 0.009) in ET by ~28% compared to SED and higher (*p* = 0.02) by 2.2% with CAF (319 ± 64 kcal) vs. no-CAF (312 ± 65 kcal). There was no group x treatment interaction (*p* = 0.91). RER was lower (*p* = 0.04) in ET (0.94 ± 0.03) vs. SED (0.98 ± 0.04). RER tended (*p* = 0.19) to be higher for CAF (0.961 ± 0.05) compared to no-CAF (0.955 ± 0.05). The %calories derived from CHO also tended to be higher (*p* = 0.07) for SED (88.5 ± 12.5%) compared to ET (79.8 ± 10.9%), but not affected by CAF (*p* = 0.13). Rate of CHO oxidation was not different (*p* = 0.09) between ET (2.34 ± 0.43 g/min) and SED (2.02 ± 0.49 g/min). For all participants, CAF elicited higher (*p* = 0.006) CHO oxidation rates (2.23 ± 0.3 g/min) vs. no-CAF (2.13 ± 0.5 g/min) and total CHO oxidized was also higher (*p* = 0.006) with CAF (66.7 ± 13.7 g) vs. no-CAF (63.9 ± 12.9 g). Rate of fat oxidation was higher (*p* = 0.03) in ET (0.27 ± 0.15 g/min) compared to SED (0.13 ± 0.16 g/min); as was total fat oxidized (8.2 ± 4.6 vs. 3.8 ± 4.2 g, respectively). CAF did not affect fat oxidation rate (*p* = 0.65) or total fat oxidized over 30 min (*p* = 0.65).

**Figure 3:**
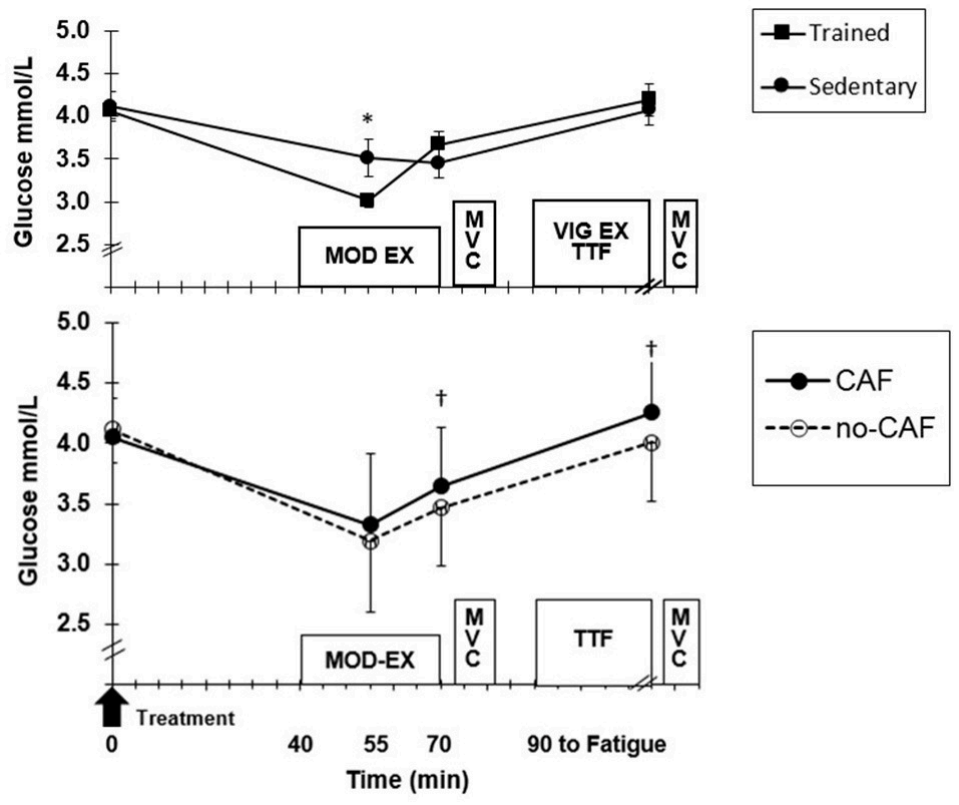
**(Top)** Mean (±SD) glucose at baseline, moderate exercise (EX) and after time to fatigue (TTF) across treatments for trained and sedentary groups. *Higher (*p* < 0.05) for sedentary. **(Bottom)** Treatment effect for all participants. ^†^Higher (*p* < 0.05) for CAF compared to no-CAF.

**Figure 4:**
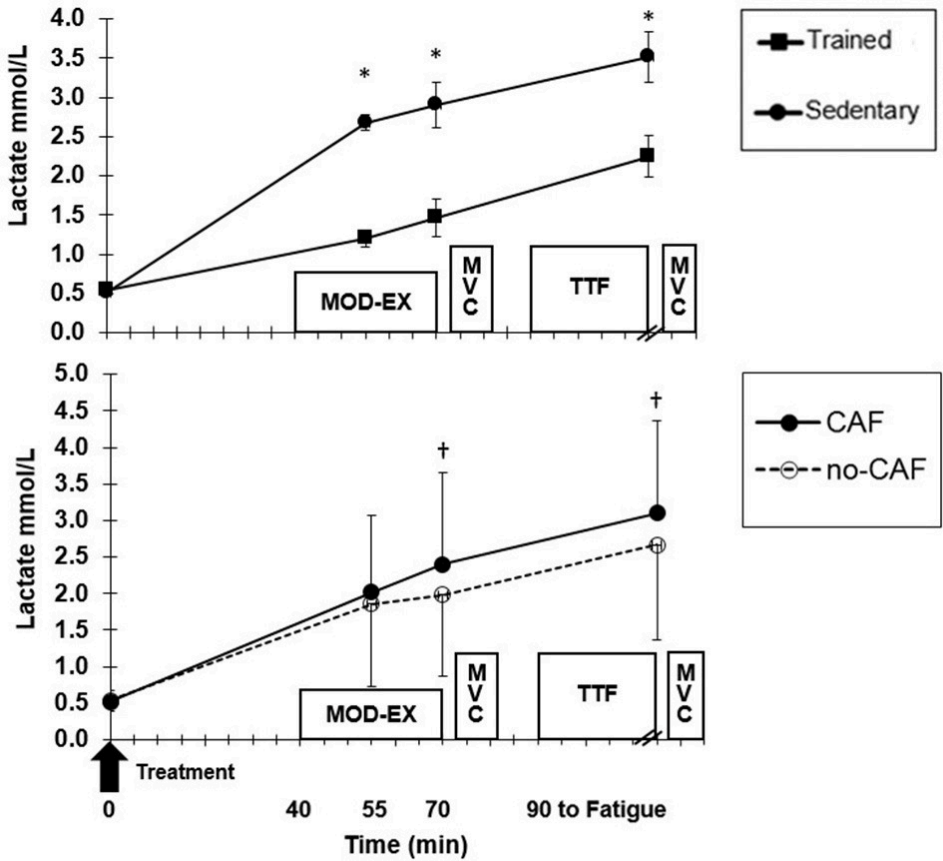
Mean (±SD) lactate at baseline, during moderate exercise (EX) and after time to fatigue (TTF). **(Top)** Trained and sedentary values for all treatments combined. *Higher (*p* < 0.01) for sedentary vs. trained. **(Bottom)** Treatment effect for all participants combined. ^†^Higher (*p* < 0.01) for CAF compared to no-CAF.

Blood glucose was not different between ET and SED, but there was a significant group x time (*p* = 0.006) interaction (Figure 3). Although ET and SED were similar at fasted rest (4.1 ± 0.2, 4.3 ± 0.3 mmol/L, respectively), ET had lower (*p* = 0.02) glucose (3.1 ± 0.6 mmol/L) at 15 min MOD-EX compared to SED (3.4 ± 0.7 mmol/L), but did not differ at the end of MOD-EX and TTF. Also, CAF resulted in higher glucose compared to no-CAF after 30 min MOD-EX (*p* = 0.04) and TTF (*p* = 0.02) (Figure 3). HLa was higher (*p* = 0.001) in SED compared to ET (Figure 4) throughout MOD-EX and after TTF (3.5 ± 0.3 vs. 2.2 ± 0.3 mmol/L). CAF elicited higher (*p* < 0.001) overall HLa during exercise (2.5 ± 0.9) vs. no-CAF (2.2 ± 0.9 mmol/L) with a treatment x time interaction (*p* = 0.007). HLa was similar up through 15 min MOD-EX but higher with CAF (*p* = 0.001) after 30 min and TTF (although participants cycled longer to fatigue with CAF).

### MVC Strength

Relative change in MVC from baseline to after MOD-EX (~ −5%) was similar (*p* = 0.09) between groups and there was no effect of CAF (*p* = 0.4). MVC strength did not decline between post MOD-EX and after TTF (*p* = 1.0), but MVC after TTF (427.1 ± 192.7 N-m) remained lower than baseline (454.6 ± 189.2 N-m, *p* = 0.007) for all participants.

## Discussion

Nutritional strategies play an influential role in attenuating fatigue during exercise. The present study verified that a moderate dose of CAF (3 mg/kg) lowered perceived effort during moderate intensity exercise and increased exercise tolerance in trained athletes but was significant by reporting a similar benefit is obtained in sedentary individuals. When consumed with a small meal prior to exercise, CAF increased CHO oxidation, blood glucose and lactate and did not augment lipid oxidation. Furthermore, ingestion of low-CHO with the meal (~40 g CHO) did not impact exercise tolerance or reduce perception of effort even though previous evidence indicated CHO mouth rinsing might improve exercise capacity in both fed and fasted states (37). Our findings collectively suggest CAF improves exercise tolerance in sedentary individuals, appearing to act primarily through central vs. peripheral mechanisms similar to endurance-trained.

These results are consistent with a meta-analysis (2) indicating CAF reduces perceived effort in trained individuals; and, that CAF facilitates greater work output in sedentary individuals without an increase in RPE (27). In the present study, CAF reduced RPE during moderate intensity exercise compared to no-CAF by ~5% and, consistent with previous findings (2, 22, 26), CAF effects on RPE were more evident in steady state exercise below LT. CAF did, however, increase exercise tolerance for both sedentary and trained participants above LT before rating the effort as “very hard.” In agreement with others (1, 31) demonstrating an ergogenic benefit for moderate doses of caffeine in trained athletes, our results indicate ~21% longer exercise capacity with CAF compared to no-CAF (ES = 0.45) for all participants. Further, the effect of CAF on time to fatigue was “small to moderate” in sedentary (ES = 0.38), consistent with the reported effect in physically fit individuals (1).

Although studies on sedentary or untrained populations are limited, there is evidence (22, 26, 27) to suggest CAF might improve exercise tolerance in this population, although not all studies have observed a benefit (24). A recent study indicates higher caffeine consumption in the diet of Japanese women was associated with higher peak oxygen consumption and moderate-to-vigorous physical activity/step counts vs. women with lower caffeine consumption, but calls for further study to clarify this association (38). One complicating factor is that the classification of “sedentary,” “untrained,” or “inactive” is not always clear among studies (30). Some define untrained at a threshold VO_2max_ or based on a survey of physical activity, without fitness level measured or reported (22–24, 26, 27). Additionally, CAF may not benefit sedentary individuals if the exercise intensity is too high (e.g., 80% VO_2peak_) (23) or during a “time trial” effort due to less experience with pacing compared to trained athletes. In contrast with the present study, CAF did not reduce RPE in sedentary females (24) during steady-state cycling or improve 10 min time trial performance. CAF improved exercise tolerance at intensities just above LT in our individuals of low fitness (VO_2peak_ < 40 ml/kg/min), although not necessarily associated with a lower RPE during the TTF which was observed in the trained participants. Different perceptual responses to CAF during exercise based on individuals' activity level has also been previously reported (25).

In cases when CAF improved exercise capacity in less fit participants (22, 27), changes in substrate oxidation are not always observed. In the present study, CAF resulted in higher blood glucose and HLa during exercise for both high and low fit participants, similar to previous reports (39, 40). CAF also increased CHO oxidation during MOD-EX, in partial agreement with others (41) reporting increased exogenous CHO oxidation during cycling. The only difference based on fitness status was higher blood glucose in our sedentary compared to trained after 15 min moderate exercise, likely as a result of ingesting the ~40 g CHO prior to exercise coupled with higher skeletal muscle glucose uptake in trained participants during exercise (42). However, this transient difference in blood glucose abated by the end of exercise. A limitation of the present study was that glycemic and insulinemic responses to the pre-exercise “feeding” and CAF beverage were not serially measured; thus, the impact of the pre-exercise feeding with or without CAF on insulin sensitivity is unclear. Previously, CAF (3–5 mg/kg) ingested 1 h prior to an oral glucose tolerance test (75 g glucose) increased insulin response and reduced sensitivity in healthy participants (28). The positive finding in our study is that if CAF *is* ingested prior to exercise, the glycemic response in sedentary mirrors that of trained individuals after 30 min of exercise.

Although trained participants had higher overall fat oxidation compared to sedentary, we did not observe a CAF effect on fat oxidation in either group, consistent with some studies (14), but not others (11, 13) reporting increased lipolysis with CAF in trained. Chronic endurance training increases capacity for fat oxidation through physiological mechanisms including increased mitochondria and capability for beta-oxidation, and greater inhibition of glycolytic enzymes. Thus, it stands to reason that sedentary individuals would have less capacity to enhance fat oxidation even with CAF. It has also been suggested (9) that trained individuals may have increased adenosine receptor sensitivity in adipose tissue compared to untrained or higher muscle sensitivity to CAF, which could imply that sedentary individuals may require a higher CAF dose (>3 mg/kg) to increase fat metabolism (12, 22).

Despite the growing popularity of caffeinated low-calorie “energy drinks,” only a few studies have compared the efficacy of CAF combined with low-CHO (25, 26) and often only vs. a placebo. Previously, we reported low-CHO ingestion attenuated mental fatigue compared to a CHO mouth rinse when fasted (43). In the present study, a small amount (< 2 g CHO in a 0.4% CHO solution) was ingested in 25 ml regular boluses during exercise along with a ~40 g CHO bar prior to exercise. Unlike CHO mouth rinse studies (which often use a higher CHO concentration drink), we did not observe an improvement in exercise tolerance using a time to fatigue test vs. PLA (ES = −0.09). When 3 mg/kg CAF was added to LCHO, there was also no added benefit compared to CAF alone (ES = 0.08). Thus, improved exercise tolerance in the present study can primarily be attributed to a moderate CAF dose and not ingestion of LCHO when in the fed state.

CAF also did not differentially influence muscular contractile properties in our trained compared to sedentary. However, neither did we observe a CAF benefit, unlike our previous findings (31, 36). MVC strength decreased in all participants after exercise but the average loss tended to be higher in SED vs. ET. CAF tended to maintain MVC strength following TTF better for ET than for SED; however, these strength data were highly variable with the inclusion of men and women of mixed fitness levels and, not all ET were cyclists as in other studies documenting MVC strength maintenance with CAF (31). The lack of CAF effect may also be attributed to the lower CAF dose and/or that our participants were not in similar states of fatigue as in previous studies (31, 44).

## Conclusion

Pre-exercise caffeine ingestion (3 mg/kg) reduces perceived effort during moderate intensity exercise and improves exercise tolerance for sedentary individuals as well as those who are highly-trained, with little difference in metabolic or physiological responses between groups. However, a low-carbohydrate drink did not provide benefit to either group following a pre-exercise feeding. Caffeine (combined with pre-exercise consumption of an energy bar) did not increase fat utilization during exercise; but, appears to increase carbohydrate oxidation, blood lactate and glycemic responses. Thus, caffeine appeared to act centrally (reducing the perception of effort). Although caffeine appears efficacious, additional research is warranted to understand the optimal dose of pre-exercise caffeine (with and without carbohydrate) for habitually sedentary individuals to improve exercise tolerance without consuming excess calories or resulting in adverse metabolic consequences.

## Ethics Statement

This study was carried out in accordance with the recommendations of the Institutional Review Board with written informed consent from all subjects. All subjects gave written informed consent in accordance with the Declaration of Helsinki. The protocol was approved by the Institutional Review Board of Georgia Institute of Technology.

## Author Contributions

NK, GW, and MM-S contributed conception and design of the study. NK organized the database. TS performed the statistical analysis. NK, GW, and MM-S wrote the first draft of the manuscript. All authors contributed to manuscript revision, read and approved the submitted version.

### Conflict of Interest Statement

The authors declare that the research was conducted in the absence of any commercial or financial relationships that could be construed as a potential conflict of interest.
